# A Review of Beef Production Systems for the Sustainable Use of Surplus Male Dairy-Origin Calves Within the UK

**DOI:** 10.3389/fvets.2021.635497

**Published:** 2021-04-27

**Authors:** Naomi H. Rutherford, Francis O. Lively, Gareth Arnott

**Affiliations:** ^1^Livestock Production Sciences Branch, Agri-Food and Biosciences Institute, Hillsborough, United Kingdom; ^2^Institute for Global Food Security, School of Biological Sciences, Queens University Belfast, Belfast, United Kingdom

**Keywords:** Holstein, steer, bull, concentrates, welfare, high input, low input, pasture

## Abstract

The UK dairy herd is predominantly of the Holstein-Friesian (HF) breed, with a major emphasis placed on milk yield. Subsequently, following years of continued single-trait selection, the beef production potential of dairy bred calves has declined. Thus, male HF calves are commonly seen as a by-product of the dairy industry. Limited markets, perceived low economic value and high rearing costs mean that these surplus calves are often euthanised shortly after birth or exported to the EU for further production. Welfare concerns have been raised regarding both euthanasia and long distance transportation of these calves. Furthermore, total UK beef consumption increased by 8.5% from 2009 to 2019. Thus, in light of this growing demand, beef from the dairy herd could be better utilized within the UK. Therefore, the potential for these calves to be used in a sustainable, cost-effective beef production system with high welfare standards within the UK requires investigation. Thus, the aim of this review was to evaluate both steer and bull beef production systems, examining the impact on performance, health, welfare, and economic potential to enable a sustainable farming practice, while meeting UK market requirements. The principal conclusions from this review indicate that there is the potential for these calves to be used in UK based production systems and meet market requirements. Of the steer production systems, a 24 month system appears to achieve a balance between input costs, growth from pasture and carcass output, albeit the literature is undecided on the optimum system. The situation is similar for bull beef production systems, high input systems do achieve the greatest gain in the shortest period of time, however, these systems are not sustainable in volatile markets with fluctuating concentrate prices. Thus, again the inclusion of a grazing period, may increase the resilience of these systems. Furthermore, production systems incorporating a period at pasture are seen to have animal welfare benefits. The main welfare concern for surplus dairy bred calves is often poor colostrum management at birth. While in steer systems, consideration needs to be given to welfare regarding castration, with the negative impacts being minimized by completing this procedure soon after birth.

## Introduction

Currently the UK dairy herd consists of 1.867 million cows (1) and is predominantly of the Holstein-Friesian (HF) breed. The main focus of the HF breed is maximizing milk yield and subsequently, following years of continued single-trait selection (2), annual milk yield per cow has increased by 14.1% in the 10 year period to 2018 (3). However, this has had a detrimental effect on other traits (e.g. functional traits, reproduction and health) and thus the beef production potential of dairy bred calves has declined, particularly in terms of carcass conformation (4, 5). Therefore, male HF calves are commonly seen as a by-product of the dairy industry (6, 7) due to limited markets and perceived low economic value (8). As a result the neonatal care of these surplus male calves is often inferior to that which heifer calves receive. Heifer calves are seen as the future of the dairy herd, and therefore, are often given priority to ensure their future productivity (9). Thus, dairy bred bull calves are at a greater risk of not receiving a sufficient quantity of quality colostrum within 24 h of birth, resulting in increased risk of failure of passive transfer (FPT) of antibodies (10). High levels of pre-wean mortality are associated with FPT, in addition to increased morbidity post-weaning, and reduced live weight gain (LWG) (11, 12).

Euthanasia soon after birth is occasionally used as a means of removing these calves from the herd, for example, in 2018 it was reported that 15% of male dairy sired calves were euthanised at birth in Great Britain (13). The high labor requirement associated with rearing, together with high rearing costs, which have been reported at £195.19 per calf from birth to weaning, mean there is little economic incentive to rear these calves when the monetary return is minimal (14, 15). This is a problem that exists worldwide. For example in New Zealand and Australia, surplus dairy calves are referred to as bobby calves and it is estimated that 2.2 million bobby calves are slaughtered for meat processing between 4 and 7 days of age in New Zealand each year (16). In the UK an alternative outlet for a large number of male dairy calves is the export market to the EU for further production such as intensive veal or bull beef. For example, in Northern Ireland a 3 year average of 19,863 calves of under 42 days of age were exported to the EU from 2015 to 2017. The vast majority of these were exported to Spain (17), where they may be finished as bull beef for the domestic market, or further exported as store cattle to non-EU countries (18). This is considered a valuable market for the industry and vastly reduces the need for euthanasia (17, 19). The live export of cattle goes beyond the UK and is a common occurrence across Europe, for example in 2017 the intra-EU live cattle trade consisted of over 3.86 million cattle, 53% of which were intended for immediate slaughter. While live cattle exported from the EU to non-EU countries was estimated to be 0.80 million cattle in 2017 (20). Unsurprisingly, animal welfare and ethical considerations have been raised regarding such live animal exports. Concerns such as journey times, animals not being given rest periods, overcrowding and poor provision of feed and water have all been highlighted (21). Research has shown that long-haul transport with deprivation of food and water can result in an increased heart rate together with implications for liver function (22). In contrast Grigor et al. (23) reported that there was no major detrimental welfare impact when calves were transported under good conditions with access to feed, water, milk replacer, although the authors did suggest that there was some negative impact on calf health status post transport. Within the UK the exporting of live animals is regulated under the Welfare of Animals (Transport) Order 2006 (24). Inspections are conducted regularly and compliance rates within the UK are reported to be high. For example in 2015 and 2016 the mean non-compliance rate for cattle was 0.06% (17). Nevertheless, live exports have received a great deal of media attention, with calls for this practice to be banned (21). Furthermore, as a result of Brexit, the UK government may now have the opportunity to impose a ban on live animal exports. Alternatively, live exports may become more heavily regulated or see additional challenges associated with market or trade agreements for transporting animals across borders (25). However, the true impact that Brexit will have is yet to be determined. In addition, the environmental impact of transporting live animals such long distances has raised concerns, with one study showing that it is more environmentally sustainable, due to reduced CO_2_ and NO_x_ emissions, to produce and slaughter lambs in their country of origin and export the carcasses, rather than operating live exports (26).

By definition in the UK, veal is meat from bovine animals slaughtered under 8 months of age. Whereas, beef is from bovine animals slaughtered over 8 months of age (27). However, in the UK the market for veal meat is small, with only 143,000 calves being slaughtered for veal in 2018 (28). Furthermore, public awareness of the product is limited and perceptions are often influenced by welfare concerns such as slaughter age (29). Therefore, the potential for these calves to be used in a sustainable, cost-effective beef production system with high welfare standards [regulated under the Welfare of Farmed Animals Regulations 2007 (30)] within their country of origin requires investigation. Here we review the literature, including examples from our own research, on the beef potential of these surplus male calves. This includes an evaluation of different production systems examining the animal performance and economic potential to enable a sustainable farming practice, while meeting the market requirements of the UK beef industry. In addition, the animal health and welfare implications are also evaluated.

## Steer Beef Production

Of the prime beef (cattle produced for the sole purpose of beef production) slaughtered in the UK in 2019, 50.9% were steers, while only 9.6% were young bulls, while the remaining 39.5% were heifers (31). A 24 months steer system, where cattle spend two seasons at pasture and are slaughtered following an indoor finishing period (32, 33), is thought to be a relatively low cost system, that gains a high proportion of growth from pasture (34). For example, Murphy et al. (35) reported HF steers growing at 0.80 and 0.93 kg/d during their first and second season at grass, respectively. These steers were finished indoors on *ad libitum* grass silage, supplemented with 5 kg dry matter (DM) of concentrate per head daily, achieving a slaughter weight, carcass weight and fat classification (on a 15 point scale) of 603 kg, 307 kg and 7.86, respectively. Similar levels of performance were achieved by McNamee et al. (36) with a LWG of 0.92 kg/d during the second grazing season and 1.45 kg/d during the finishing period [concentrates fed at 67% of dry matter intake (DMI) during finishing]. A summary of the key findings on steer finishing performance from published literature are presented in Table 1 however, the literature assessing Holstein steer production systems is relatively limited.

**Table 1:** Summary of key published research^a^ addressing the effect of finishing diet on steer performance.

**Finishing diet**	**Slaughter age (months)**	**Finishing LWG (kg/d)**	**Slaughter weight (kg)**	**Carcass weight (kg)**	**Breed**	**References**
Pasture only for 94 days	21	0.82	496	244	Holstein-Friesian, Aberdeen Angus × Holstein-Friesian & Belgian Blue × Holstein-Friesian^c^	(37)
Pasture only for 94 days then silage + *ad libitum* concentrates for 98 days	24	1.33	627	329	Holstein-Friesian, Aberdeen Angus × Holstein-Friesian & Belgian Blue × Holstein-Friesian^c^	(37)
Pasture + 5 kgDM/d concentrate for 60 days	21	0.90	535	275	Holstein-Friesian	(38)
Pasture + 5 kgDM/d concentrates for 68 days	21	1.11	535	277	Holstein-Friesian	(35)
Pasture + 5 kgDM/d concentrate for 110 days	21	0.99	537	276	Holstein-Friesian	(38)
Silage + 5 kgDM/d concentrate for 92 days	24	0.97	603	307	Holstein-Friesian	(35)
Silage + 5 kgDM/d concentrate for 92 days	24	0.91	612	308	Holstein-Friesian	(38)
Total mixed ration of 0.67 concentrates and 0.33 silage (on a DM basis)^b^	25	1.45	594	289	Holstein-Friesian, Norwegian Red × Holstein-Friesian & Jersey × Holstein-Friesian^c^	(36)
Silage + *ad libitum* concentrate for 94 days	21	1.49	551	287	Holstein-Friesian, Aberdeen Angus × Holstein-Friesian & Belgian Blue × Holstein-Friesian^c^	(37)

*LWG, live weight gain; DM, dry matter*.

a *Treatments or studies were steers have been slaughtered over 30 months of age have not been included*.

b *Finishing duration not reported*.

c *These studies only reported the main effects of breed and production system*.

*The databases used in this literature search were Web of Science, Scopus, and Google Scholar*.

Alternative production systems for steers involve earlier finishing, usually off pasture at the end of the second grazing season. Keane and Moloney (37) compared two early (~20 months) finishing strategies to the traditional 24 months system. The authors reported that finishing steers early lead to insufficient carcass weights and fat cover; while, *ad libitum* concentrates during the finishing period of the early strategy proved to be uneconomical. Thus, the most successful of the three systems was deemed to be the 24 months system when the grazing period was followed by a 3 months *ad libitum* finishing period; this system resulted in a lower concentrate intake per kg live weight (LW) than early *ad libitum* finishing (37). Murphy et al. (38), conducted a similar study and also found fat cover to be lower in a 21 months pasture finishing systems compared to a 24 months system. The reduced fat deposition at 21 months may be a limitation of implementing this system with the HF breed, due to their later maturing nature. Thus, a 21 months system may be more suitable for early maturing × dairy bred cattle such as Aberdeen Angus (37). Murphy et al. (38) also reported that slaughtering at 21 months following either a 60 or 120 days 5 kgDM concentrate supplementation period produced similar carcass weights of 275 and 276 kg, respectively, in comparison to 308 kg for a 24 months system (also offered 5 kgDM concentrate supplementation during indoor finishing). However, in contrast to Keane and Moloney (37) and Murphy et al. (38) reported that a 21 months system with a 60 days concentrate finishing period at pasture resulted in the greatest net profit margin (€55/head), while the 24 months steer system actually resulted in a net loss (–€29/head). Murphy et al. (38) took into consideration the additional land charge associated with keeping 24 months steers for an additional 3 months. The authors reported that the additional carcass gain achieved during this time was not sufficient to sustain the additional costs associated with land and feed. With feed costs accounting for a substantial proportion (75%) of the variable costs in beef production (39), it is important that a balance is reached between production costs and carcass output. Overall, the literature indicates that carcass output, concentrate costs, and stocking rate will largely determine the most economical steer beef production system. The environmental impact of a shorter steer system has also been considered. In the case of Murphy et al. (38) finishing steers at pasture with 60 days of concentrates resulted in an 11% reduction in GHG emissions per kg beef produced compared to a 24 months system. Yet, when the unit of measure is changed to GHG emissions per hectare that of the 21 months system is greater due to the greater stocking rate associated with the shorter production cycle.

UK steer market requirements dictate a maximum age of 30 months. However, the literature on Holstein steer systems largely focuses on slaughtering cattle at 21 and 24 months, and thus does not consider more extensive systems. Keane and Allen (40) compared an extensive (29 months) system to a conventional (24 months) system with Charolais × Friesian steers. Due to the study design, cattle were slaughtered at a target live weight and thus, carcass weights were consistent across the two systems. Interestingly, the authors summarized the inputs and outputs of the two systems, reporting that the extensive system had a greater gross profit margin per head [156 vs. 34 European Currency Unit (ECU)] and per hectare (229 vs. 71 ECU) than the conventional system. The lower production costs associated with the reduced concentrate intake in the extensive system was the main contributing factor (40). Thus, extensive production systems may have some merit as a Holstein beef system. However, from an environmental perspective further increasing the age at slaughter would be expected to result in a greater GHG output per head and per kg beef (35). With recent Net-Zero Carbon targets outlined, the livestock sector will have to consider both the economic and environmental sustainability of its production systems going forward (41).

## Bull Beef Production

Characteristically, bulls have a much higher growth rate potential and improved feed efficiency than steers (6, 42, 43). Under UK market requirements, bulls are to be slaughtered under 16 months of age, with research demonstrating that the slaughter age of bulls will directly impact on a number of meat eating qualities (44). Bulls slaughtered outside of market specification will be subject to reduction in the price paid per kg carcass. These market requirements create both challenges and opportunities for bull beef production. In order to achieve desired carcass weights at 16 months, there is little room for setbacks in LWG during the production cycle (45). Bearing in mind that male dairy bred calves often have an inferior health status (10), this can result in longer term indirect effects associated with reduced lung function, feed efficiency and LWG (46). Therefore, ensuring target weights are achieved throughout the production system can often be a challenge for producers. One potential advantage of the current market requirements is that the shorter production cycle for bull beef results in a faster throughput of cattle on farm. This is particularly advantageous where the availability of land or housing are limiting factors. Furthermore, a 16 months bull beef system has been reported to result in lower greenhouse gas emissions, than a steer system, due to slaughtering animals at a younger age (35, 47). However, if the bull beef system relies on imported feed this can substantially increase greenhouse gas emissions in comparison to one which uses nationally or home grown feedstuffs (48). The key findings from published bull beef research are summarized in Table 2 and these will be discussed in the following three sub-sections.

**Table 2:** Summary of key published research^a^ addressing the effect of production system on bull performance and concentrate intake.

**Growing diet**	**Growing LWG (kg/d)**	**Finishing diet**	**Slaughter age (months)**	**Finishing LWG (kg/d)**	**Slaughter weight (kg)**	**Carcass weight (kg)**	**Total concentrates^b^ (kgDM)**	**Breed**	**References**
Pasture	1.11	Straw + *ad libitum* concentrates for 69 days	14	1.21	455	238	–	Danish Friesian	(49)
Pasture (autumn born bulls)	0.72	Silage + *ad libitum* concentrates for 191 days	15.5	1.61	581	291	1,700	Holstein	(50)
Pasture	0.90	Straw + *ad libitum* barley based concentrate for 209 days	15	1.56	546	283	1,673	Holstein-Friesian	(45)
Pasture (spring born bulls)	0.66	Silage + *ad libitum* concentrates for 209 days	15.5	1.38	510	258	1,440	Holstein	(50)
Pasture + 1 kgDM/d concentrates	0.87	Silage + *ad libitum* concentrates for 200 days	15	1.56	542	280	1,602	Holstein-Friesian	(35)
Pasture + 2 kgFW/d concentrates (autumn born bulls)	0.99	Silage + *ad libitum* concentrates for 191 days	15.5	1.48	575	295	1,790	Holstein	(50)
Pasture + 2 kgFW/d concentrates (spring born bulls)	0.87	Silage + *ad libitum* concentrates for 209 days	15.5	1.43	548	281	1,840	Holstein	(50)
Pasture + 3 kgDM/d barley + maize based concentrate	1.10	Straw + *ad libitum* barley based concentrate with rumen protected fat for 209 days	15	1.40	552	281	1,866	Holstein-Friesian	(45)
Pasture + 3 kgDM/d barley based concentrate	1.07	Straw + *ad libitum* barley + maize based concentrate for 209 days	15	1.47	554	288	1,578	Holstein-Friesian	(45)
Pasture + 3 kgDM/d barley based concentrate	0.96	Straw + *ad libitum* barley based concentrate for 209 days	15	1.55	572	296	1,774	Holstein-Friesian	(45)
Pasture + *ad libitum* concentrates (autumn born bulls)	1.38	Silage + *ad libitum* concentrates for 191 days	15.5	1.46	615	315	2,350	Holstein	(50)
Pasture + *ad libitum* concentrates (spring born bulls)	1.27	Silage + *ad libitum* concentrates for 209 days	15.5	1.28	579	296	2,330	Holstein	(50)
Silage + *ad libitum* concentrates (autumn born bulls)	1.58	Silage + *ad libitum* concentrates for 191 days	15.5	1.29	600	313	2,240	Holstein	(50)
Silage + *ad libitum* concentrates (spring born bulls)	1.34	Silage + *ad libitum* concentrates for 209 days	15.5	1.22	579	296	2,200	Holstein	(50)
–	–	Pasture + *ad libitum* concentrates for 180 days	12	1.31	473	246	–	Holstein-Friesian	(51)
–	–	Silage + low concentrate level (decreasing) for 438 days	16	1.02	539	278	665	Finnish Ayrshire	(52)
–	–	Silage + low concentrate level (constant) for 424 days	16	1.11	558	285	640	Finnish Ayrshire	(52)
–	–	Silage + low concentrate level (increasing) for 440 days	16	1.05	554	291	677	Finnish Ayrshire	(52)
–	–	50:50 silage:concentrate for 308 days	16	1.14	545	279	1,191	Norwegian Red & Holstein-Friesian^c^	(5)
–	–	Silage + high concentrate level (decreasing) for 402 days	16	1.16	552	290	1,193	Finnish Ayrshire	(52)
–	–	Silage + high concentrate level (constant) for 404 days	16	1.16	558	297	1,187	Finnish Ayrshire	(52)
–	–	Silage + high concentrate level (increasing) for 409 days	16	1.16	565	299	1,116	Finnish Ayrshire	(52)
–	–	Straw + *ad libitum* concentrates for 135 days	8	1.32	300	155	624	Holstein	(6)
–	–	Straw + *ad libitum* concentrates for 177 days	9.5	1.27	350	179	876	Holstein	(6)
–	–	Straw + *ad libitum* concentrates for 180 days	12	1.24	541	235	–	Holstein-Friesian	(51)
–	–	Straw + *ad libitum* concentrates for 197 days	10	1.39	400	211	1,111	Holstein	(6)
–	–	Straw + *ad libitum* concentrates for 239 days	11.5	1.36	450	237	1,429	Holstein	(6)
–	–	Straw + *ad libitum* concentrates for 285 days	13	1.31	500	265	1,779	Holstein	(6)
–	–	Straw + *ad libitum* concentrates for 322 days	14	1.33	550	294	2,131	Holstein	(6)

*LWG, live weight gain; DM, dry matter*.

a *Treatments or studies were bulls have been slaughtered over 16 months of age have not been included*.

b *The amount reported here is the total concentrates fed over the full duration of each study*.

c *This study only reported the main effects of breed and production system*.

*The databases used in this literature search were Web of Science, Scopus, and Google Scholar*.

### High Input Bull Beef Production Systems

Bull beef production is traditionally an intensive *ad libitum* concentrate, indoor system (6, 53). *Ad libitum* feeding has the potential to lead to superior weight gains by supporting the genetic potential of the animal. Supplementing concentrates at 95% of DMI from 3 months of age until slaughter has been shown to result in a mean LWG of 1.33 kg/d, with bulls reaching target slaughter weight (550 kgLW) at 14 months of age (6). Rutherford et al. (50) offered housed autumn born Holstein bulls *ad libitum* concentrates from 6 months until slaughter at 15.5 months and achieved a mean LWG of 1.39 kg and carcass weight of 313.3 kg. These results are in agreement with previous *ad libitum* studies (49, 51) and show the potential for bulls to achieve a high level of performance when offered an intensive diet.

The literature has also explored the effect of *ad libitum* concentrates at grass. Rutherford et al. (50) offered bulls *ad libitum* concentrates at grass during the summer followed by a housed *ad libitum* finishing period. This system resulted in a 1.44 kg/d LWG and 314.5 kg carcass weight at 15.5 months. Similarly, Moloney et al. (51) slaughtered bulls at ~12 months of age, following a 6 months period of *ad libitum* concentrates at grass achieving a LWG and carcass weight of 1.31 kg/d and 245.6 kg, respectively. The lighter carcass weight here, being a result of the younger age at slaughter. It was also reported that total concentrate intake did not differ between bulls that were housed or grazed with *ad libitum* concentrates (50). Furthermore, in both studies meat quality parameters were unaffected by *ad libitum* production system (50, 51). Although the forage component of the diet differed, both production systems were heavily dependent on concentrates and thus the energy dense diet resulted in sufficient muscle and liver glycogen stores for anaerobic glycolysis to occur post-mortem (54, 55).

One limitation of an *ad libitum* concentrate system, is that it often results in a high cost of production due to the high input nature of the diet. For example, total concentrate consumption from *ad libitum* production systems has been reported at 2.13 tDM (6) and 2.34 tDM (50) during the study period. This therefore creates a system that is subject to fluctuating concentrate prices and beef market volatility (34) and thus profit margins can vary substantially from year to year. In addition, a high input, housed system, may also raise animal health and welfare concerns which are discussed in section Effect of Beef Production System on Health and Welfare.

### Housed Low Input Bull Beef Production Systems

When combined with good quality silage, moderate (56) or no (57) concentrate supplementation can support high levels of performance. Moving away from offering *ad libitum* concentrates and therefore, reducing the dependence on them, has the potential to lessen the cost of production. Kirkland et al. (5) offered concentrates at 50% of DMI (mean of 3.87 kgDM/d) from 6 to 16 months of age; achieving a LWG of 1.14 kg/d carcass weight of 278.8 kg. During the 249 d experimental period, total concentrate intake equated to 1.14 tDM (5); considerably lower than that of *ad libitum* fed bulls (6, 50).

Manni et al. (52) achieved a LWG of 1.16 kg/d from Finnish Ayrshire bulls when supplementing concentrates at 42% DMI. While a concentrate inclusion rate of 23% DMI resulted in a slightly lower LWG of 1.11 kg/d. The lower concentrate also meant that these bulls required an additional 20 days to reach target slaughter weight. Even though both groups were slaughtered at the same LW (558 kg), differences were observed in carcass fatness, with the bulls on the higher concentrate inclusion rate being fatter. This indicates that the greater proportion of metabolisable energy in the diet from concentrates, not only supported a greater LWG but resulted in increased fat deposition (52), which as previously stated can have a detrimental effect on feed efficiency (58). However, fat cover is considered an important component of meat eating quality, as intramuscular fat can lead to increased flavor and tenderness of the meat (59).

Research has also investigated finishing dairy bulls up to 20 months of age. Under the current UK market requirements, these systems would not produce beef that is within market specification, however, some interesting trends were reported that could be used to inform a 16 months system. Although increasing slaughter age often results in a heavier carcass weight, growth rates have been shown to slow in the final months prior to slaughter. This was evident in the study conducted by Kirkland et al. (5) where increasing slaughter age from 16 to 20 months resulted in an increased LW at slaughter of 111 kg. However, growth rates were compromised during this additional 4 months of finishing. Bulls slaughtered at 20 months had a mean LWG of 1.14 kg/d up to 16 months of age; following this, mean LWG decreased to 0.88 kg/d; a substantial 30% reduction (5). This trend had previously been reported (60, 61) and reflects the sigmoidal growth curve of cattle (62).

### Grazed Bull Beef Production Systems

Profitability in a dairy-origin beef production system is reported to be determined by two main factors; carcass output per hectare and the proportion of grazed grass in the diet (63). Grazed grass is known to be the cheapest form of feed for ruminant production (33, 64). Thus, the inclusion of a grazing period during the production system, may have the potential to reduce production costs considerably. However, managing cattle in a rotational pasture system can be more labor intensive than a housed system, particularly if the grazing infrastructure is suboptimal. The most suitable time for a grazing period in bull beef production is during the grower period, to allow for an indoor finishing period so that carcass traits are not compromised.

A LWG from grass of 0.72 and 0.80 kg/d have been reported during a 203 and 134 d grazing period, respectively for HF bulls (65). Therkildsen et al. (49) observed a greater LWG of 1.1 kg/d from grass for Danish Friesian bulls from 7 to 12 months of age. However, this was still 37% lower than that of housed *ad libitum* concentrate bulls. Supplementing concentrates during the grazing period may aid in maintaining a greater level of performance. Offering bulls 2 kg/d concentrates while at grass has been shown to support a LWG of 0.99 kg/d for autumn born bulls and 0.87 kg/d for spring born bulls. This was greater than that achieved from grass alone in the same study; 0.72 kg/d for autumn born bulls and 0.66 kg/d for spring born bulls (50).

Herbage quality (45) and pre-grazing height (66) are said to be two of the main factors influencing the LWG achievable from grass. Reducing sward-surface height from 10.0 to 6.5 cm resulted in 0.33 kg/d reduction in LWG in Continental × Friesian bulls (66). In addition, grazing weaned Friesian (FR) bulls on herb-legume swards has been reported to result in greater performance in comparison to grazing pasture or pasture with concentrate supplementation (67). However, this study focused purely on the summer grazing period, and thus it is unknown as to whether the live weight advantage continued throughout the finishing period. Therefore, the literature is limited on the effect of different pasture types in Holstein bull beef production systems from post-weaning to slaughter, thus this is an area that warrants further research.

One of the challenges of grazing is that a post-turnout LW loss can occur when calves are turned out to pasture. In the case of Steen and Kilpatrick (66) this resulted in a reduction in LW of up to 15 kg over a period of 2 weeks. This reduces the efficiency and LWG achieved from grass, as the period of loss and subsequent recovery time result in a period of unproductiveness. However, the extent of this can be reduced if cattle are fed a high forage diet pre-turnout, as opposed to having a high level of concentrate supplementation (68) and grazing and weather conditions are good at turnout. Other problems can arise with bulls at pasture, particularly if weather conditions are not optimal. Bulls are renowned for agonistic and sexual behavior (69), and being generally more unsettled that steers. The likelihood of which is amplified if conditions are wet, bulls are grazed in large groups or have a lack of shelter in a paddock grazing system (70). This can lead to increased poaching and a reduction in the grazable area. Thus, early housing of bulls in the autumn can reduce setbacks in LWG (65) and sward damage. Adverse grazing conditions were thought to be largely responsible for the poor LWG (0.58 kg/d) of bulls during the grazing season observed by McNamee et al. (36). Keane and Fallon (65) reported that bulls housed in early September were of better body condition than those housed in late November. This was confirmed by a reduction in mean LWG from grass of 0.08 kg/d when the grazing season was extended. Considering the relatively short duration for finishing following a summer grazing period (particularly for autumn born bulls) it is vital that a balance is reached between maximizing the inclusion of grass in the diet and LW performance.

Once housed, the level of concentrate feeding during the finishing period must be carefully considered. Depending on the length of the grazing period; bulls are likely to be considerably lighter than if they had been housed on an *ad libitum* system (6, 49, 50, 65). Thus, in order to ensure carcass weight is not substantially compromised, the aim should be to maximize carcass gain during the subsequent finishing period. Where concentrate supplementation is not offered during the grazing period, a finishing period of at least 200 days is said to be required (71).

Following a grazing period, Rutherford et al. (50) offered autumn born bulls *ad libitum* concentrates during finishing, achieving a LWG of 1.32 kg/d and carcass weight of 291.3 kg, which was 22 kg less than that achieved by lifetime *ad libitum* bulls. Thus, it was suggested that these bulls expressed compensatory growth during the finishing period (72). Furthermore, Rutherford et al. (50) reported similar carcass weights between bulls offered 0 and 2 kg/d concentrates at grass during the grower period. This was in agreement with Murphy et al. (45) who also reported similar finishing LWGs and carcass weights for bulls supplemented and unsupplemented at grass. Furthermore, mean carcass fat classification did not differ between these groups. Yet when the authors considered the proportion of bulls in each treatment that achieved the target fat classification, those that had received 3 kg/d concentrates at grass, were proportionately 40% more likely to reach target fat classification than those unsupplemented (45).

Keane and Fallon (65) evaluated three levels of concentrate feeding (3, 6 kg/d and *ad libitum*) for finishing HF bulls following a grazing period. A*d libitum* concentrate feeding achieved a mean LWG of 1.36 kg/d; leading to a LW at slaughter of 610 kg. These bulls obtained a carcass weight of 338 kg, and fat classification of 3.2 following a mean finishing period of 225 days. Whereas, those on 3 kg/d concentrates had a LWG of 0.91 kg/d, and LW at slaughter of 514 kg. Slaughter traits such as dressing proportion, carcass conformation, fat classification were also reduced as a result of the lighter slaughter weight (65). Although not specified, it is estimated that these bulls were approaching 18 months at slaughter, and thus alterations would need to be made to meet current market requirements.

The finishing diet must take into account the total feed input, the duration of finishing and the desired level of carcass finish. Keane and Fallon (65) concluded that commencing a finishing period at 300 kgLW following a grazing period, would require 1.9 tDM of concentrates and 0.2 tDM of silage over a 7 months period when concentrates were supplemented *ad libitum*, to achieve a carcass weight of 320 kg. A further 1.5 months of finishing was required when concentrates were supplemented at 6 kgDM/d; equalling a total concentrate intake during the finishing period of 1.3 tDM along with a 1.1 tDM silage intake. Thus, a balance between feed input and length of finishing period needs to be considered when identifying the optimum production system for bull beef. This will largely be determined by the age and weight of the bulls at the start of the finishing period. If carcass weights are to be lighter as a result of including a grazing period, then a lower cost of production must be guaranteed. Rutherford et al. (50) demonstrated that the inclusion of a grazing period could reduce total concentrate usage by 890 kg in spring born bulls compared to a housed *ad libitum* concentrate system, thus substantially reducing production costs. Therefore, although careful management is required to ensure market requirements are achieved (45), the literature does suggest that the inclusion of a grazing period in a bull beef system is a potential method of operating a sustainable bull beef production system.

## Effect of Beef Production System on Health and Welfare

One of the main welfare concerns in a steer production system is the age and method of castration. Castration is well-known to cause pain and elicit a stress response, illustrated by a rise in plasma cortisol concentration and changes in calf behavior (73). Furthermore, neurohormonal and electroencephalographic stress responses to castration have been shown to be age-specific (74). Therefore, the method and age of castration of calves is controlled under UK legislation [the Protection of Animals (Anesthetic) Act 1954 and the Veterinary Surgeons Act 1966] (75), in order to minimize the negative impact on animal welfare. For example, the use of rubber ring castration can only be completed up to 7 days of age, whereas for calves up to 2 months of age, bloodless castration (crushing the spermatic cords) is the recommended method. After 2 months of age castration should be carried out be a veterinary surgeon (75). From a production perspective, it is recommended that calves are castrated at birth or close to birth, as live weight loss increases quadratically as the age of castration increases, regardless of the method used (76).

Behavioral freedom is an important component of animal welfare, and pasture based production systems are perceived to offer this to a greater extent than continuously housed systems (77). Research in dairy cows has shown that cows at pasture will spend a greater amount of time lying (78) and feeding (79) than those housed. Furthermore, bulls that have been housed throughout their lifetime have been observed to be more aggressive toward humans and one another, compared to those that have been at pasture (66). Therefore, a pasture based steer system or bull beef system with the inclusion of a grazing period, would be considered best in providing behavioral freedom for beef cattle, as this way cattle spend a lesser proportion of their lifetime housed. However, the handling and feeding of bulls at pasture can be a potential safety risk for the farmer, and thus care must be taken when carrying out these tasks. In contrast, the use of well-designed housing and handling facilities, make feeding and routine procedures such as weighing easier and safer to complete in a housed system. Yet regardless of whether bulls are produced indoors or at pasture there will always be an increased safety risk when working with bulls as opposed to steers.

Another consideration of continuously housed beef systems is the impact on lameness. Lameness has a multifactorial and complex etiology (77), and research on beef production systems has largely focused on the impacts of different floor surfaces (concrete, rubber, and straw) in housed systems (80, 81). Slatted concrete flooring has been documented to be a contributing factor, increase the incidence and severity of sole hemorrhages in dairy bred calves in comparison to straw bedding (80). Yet little is known about the impacts of a fully housed beef system in comparison to a pasture based beef system.

Subacute ruminal acidosis (SARA) is a common nutritional disease in beef production, which can have a considerable detrimental effect on animal health, welfare, and performance (82–84). The primary cause of SARA is the consumption of excessive amounts of rapidly fermentable carbohydrates in conjunction with inadequate fiber (85); resulting in an increased accumulation of organic acids within the rumen. The effects of which are compounded if the rumen environment is not sufficiently adapted to concentrate feeding (85). Thus, SARA is a health risk of intensive production systems, such as an *ad libitum* concentrate bull beef systems, or those where an appropriate transition period is not adhered to at the beginning of the finishing period. Liver abscesses are often a secondary outcome of SARA, and may result in a further reduction in DMI, LWG, feed efficiency and carcass yield (86). However, in the case of Rutherford et al. (50) bull beef production system had no impact on the post-mortem incidence of liver abscesses, thus indicating that with the appropriate management, this can be avoided.

Other parameters such as clinical pneumonia incidence, together with the post-mortem incidence of pneumonia and pericarditis have also been considered and found to be unaffected by production system (50). Similarly, Manni et al. (87) also reported no clinical signs of disease during a bull beef production system study in Finland.

Bulls are reported to be more susceptible to stress than steers (88). Thus, pre-slaughter stressors associated with handling, re-grouping, transportation, and lairage (89, 90) should be minimized in order to reduce the risk to both animal welfare and meat quality (91, 92). Through the use of rumen temperature boluses, Rutherford et al. (93) demonstrated that bulls that displayed stress induced hyperthermia during the pre-slaughter phase also had the poorest meat quality. Suboptimal meat quality can result in a substantial cost to the red meat industry (94), thus bull beef systems may require better pre-slaughter management than that of steer systems.

The literature on beef production systems rarely evaluates the impacts on animal health and/or welfare, as the primary focus of these studies are intakes, performance, and carcass characteristics. Thus, direct comparisons of the welfare of beef production systems are particularly limited within the scientific literature. Therefore, future research evaluating these systems should involve a much wider assessment, beyond simply the impacts on animal productivity.

## Conclusion

This review demonstrates that there is the potential for surplus male dairy bred calves to be used in UK beef production and meet market requirements. Whether the system implemented involves steers or bulls is something that will vary from farm to farm. Profitability is highly subject to fluctuations in calf, concentrate and beef price, while a high carcass output per hectare combined with a low total concentrate intake, have been shown to be key measures within a production system that can be farmer managed to maximize the sustainability of a production system (34, 35). Thus, it would be expected that the most suitable systems are those that achieve a high proportion of growth from pasture, such as a 24 months steer system, or a bull beef system that involves a grazing period.

A number of health and welfare challenges associated with beef production systems were highlighted within this review. However, the castration of calves is regulated within the UK, while health issues such as SARA can be prevented with appropriate nutritional management. Thus, these are challenges that can be overcome and do not raise the same welfare concerns as the current methods of exporting or euthanising surplus calves.

The issue of surplus male dairy calves may also be addressed *via* breeding programmes within the UK dairy herd. The increased use of sexed semen for targeted breeding of dairy replacements (95), now means that more beef genetics are being used within dairy herds (96). Beef sired dairy calves are well-known to have improved carcass gain and carcass characteristics while also having a lower DMI than their dairy sired counterparts (97) and thus are better suited for beef production.

Beef from the dairy herd has also been shown to have significantly less GHG emissions than that from the suckler herd (35, 98) and therefore from an environmental perspective surplus male calves should be utilized to their full potential within the UK. However, one consideration of using surplus dairy bred calves in UK beef production is to ensure that this additional beef production would not flood the market and drive prices down. However, the UK produces beef to some of the highest welfare standards in the world, in addition to a large proportion of beef being grass fed (33), and thus the marketing potential of this product should assist in sourcing and expanding markets.

## Author Contributions

NR: writing—original draft preparation. NR, GA, and FL: writing—review and editing. All authors: contributed to the article and approved the submitted version.

## Conflict of Interest

The authors declare that the research was conducted in the absence of any commercial or financial relationships that could be construed as a potential conflict of interest.
